# The effect of the novel phosphodiesterase-4 inhibitor MEM 1414 on the allergen induced responses in mild asthma

**DOI:** 10.1186/1471-2466-14-166

**Published:** 2014-10-28

**Authors:** Brian R Leaker, Dave Singh, Ferhana Y Ali, Peter J Barnes, Brian O’Connor

**Affiliations:** Respiratory Clinical Trials Ltd, 20 Queen Anne Street, London, W1G 8HU UK; Medicines Evaluation Unit, University Hospital of South Manchester Foundation Trust, University of Manchester, Manchester, UK; National Heart & Lung Institute, Imperial College, London, SW3 6LY UK

**Keywords:** Phosphodiesterase (PDE4), Inhaled allergen challenge, Asthma, COPD, Biomarkers, TNFα, LTB_4_

## Abstract

**Background:**

Inhaled allergen challenge is a standard method to study airway responses to inflammatory provocation and evaluate the therapeutic potential of novel anti-inflammatory compounds in asthma. MEM 1414 is a novel oral PDE4 inhibitor with high affinity and selectivity creating the potential for an improved side effect profile vs non-selective PDE inhibitors. We evaluated the tolerability and effect of MEM 1414 on airway responses in mild asthmatics.

**Methods:**

A randomised double blind placebo controlled cross over study in two centres, in which sixteen steroid naïve atopic asthmatics were challenged with inhaled allergen. Subjects were dosed with MEM 1414 (600 mg) or placebo, twice daily orally for 7 days. Allergen challenge was performed on day 6 (2 hours post-dose), and methacholine responsiveness was measured 24 hours post allergen (day 7). Biomarkers of drug effects using *ex vivo* LPS stimulation of whole blood production of interleukin (IL)-6 and leukotriene (LT)-B4 and fractional exhaled nitric oxide (FeNO) were measured on day 6 (0, 2 and 8 hours post-dose). Plasma pharmacokinetics were measured on days 1, 6 and 7. The primary endpoint was the effect on late asthmatic response to allergen.

**Results:**

Treatment with MEM 1414 abrogated the late phase response with a mean difference in FEV_1_ (LAR 3–10 hours) of 104 ml (25%) vs placebo (p < 0.005), with no effect on the early response. Biomarker responses were also attenuated with MEM 1414 treatment with reductions in LPS-stimulated whole blood assays for TNFα at 8 hours (p < 0.03) and LTB_4_ at 24 hours (p = 0.0808) with no change in the IL-6 response. The MEM 1414 treatment phase was associated with higher incidence of nausea (6/16 MEM 1414 vs 2/16 placebo) and vomiting (3/16 vs 0/16 placebo).

**Conclusions:**

Oral MEM 1414, a novel PDE4 inhibitor, significantly reduces the late response following inhaled allergen challenge. MEM 1414 also inhibited whole blood assays of cytokine production from inflammatory cells. MEM 1414 was associated with a typical adverse event profile of PDE4 inhibitors, namely nausea and vomiting although these were mild side effects.

**Trial registration number:**

Current controlled trials ISRCTN48047493.

## Background

Although inhaled glucocorticoids are the mainstay of current asthma therapy there remains a considerable group of patients with poor symptom control [1] which has led to the search for novel anti-inflammatory therapies. The non-selective oral phosphodiesterase (PDE) inhibitor theophylline has been used as a treatment for asthma for many years. However, it has a low therapeutic index due to limited potency and a poor side effect profile [2, 3]. Selective PDE4 inhibitors have recently been developed with the aim of improving the therapeutic index, as the PDE4 isoform is highly expressed on inflammatory cells involved in asthma and COPD, such as mast cells, eosinophils, T lymphocytes, macrophages and neutrophils [4, 5]. PDE4 inhibition reduces breakdown of cyclic 3’5’-adenosine monophosphate (cAMP) and increases phosphorylation of intracellular proteins, resulting in *in vivo* effects including smooth muscle relaxation and suppression of immune cell function [5, 6].

Animal models have shown this approach to be highly effective in reducing allergen induced inflammation [7, 8]. Clinical studies have also shown efficacy for other orally administered PDE4 selective inhibitors on relevant asthma endpoints such as inhibition of allergen challenge [9, 10] and exercise induced bronchoconstriction [11], as well as improvements in lung function [12]. However, the tolerability of these orally administered drugs is limited by side effects such as gastro-intestinal symptoms [13–15].

MEM 1414 was screened with a standard panel of over 75 binding sites for receptors and ion channels, and 30 enzymes. MEM 1414 was found to be highly selective for the PDE4 binding site and selectively inhibited PDE4 enzyme activity. MEM 1414 has nanomolar affinity, and high selectivity for PDE4 over other PDEs such as 1, 2, 3, 5, 6 and 7, and shows efficacy in animal models of pulmonary inflammation (data on file, Memory Pharmaceuticals Corp.). In human recombinant enzyme inhibition assays the IC50 values for MEM 1414 on PDE4A, PDE4B and PDE4D were 22 nM, 18 nM and 12 nM respectively, demonstrating that MEM 1414 is a non-selective PDE4 inhibitor. These values are similar to and comparable with the non-selective PDE4 inhibitor roflumilast which display IC50 values of 0.9, 0.2 and 0.4 nM for PDE4A, PDE4B and PDE4D respectively [16]. *In vivo* data from rat models found MEM 1414 at 30 and 100 mg/kg orally inhibited LPS-stimulated neutrophil lung infiltration. MEM 1414, at 30 and 100 mg/kg, reduced the number of eosinophils in rat lung 48 hours after exposure to ovalbumin. LPS-stimulated TNFα production in whole blood was inhibited by doses of MEM 1414 from 0.1 to 100 mg/kg in rats. A dose of 30 mg/kg MEM 1414 *i.p.* significantly increased cAMP levels in rat hippocampus, consistent with its proposed method of action. These pre-clinical data suggest that MEM 1414 may be an effective and selective PDE4 inhibitor with a potent anti-inflammatory effect. The aim of this proof of concept study was to investigate the effects of MEM 1414 on allergen-induced responses in subjects with mild asthma.

## Methods

### Subjects

Sixteen steroid naïve subjects with a confirmed diagnosis of asthma for at least 6 months were recruited. Subjects were aged between 18 to 55 years and non-smokers. At screening, subjects were required to have a forced expiratory volume in 1 second (FEV_1_) >75% predicted, have a positive skin test to either house dust mite, grass pollen or cat allergen, and to demonstrate both an early and late asthmatic reaction to one of these allergens when inhaled. All subjects provided written informed consent. The study was approved by London Research Ethics Committee (now called Brent REC); reference number 08/H0718/73.

### Study design

**Figure 1:**
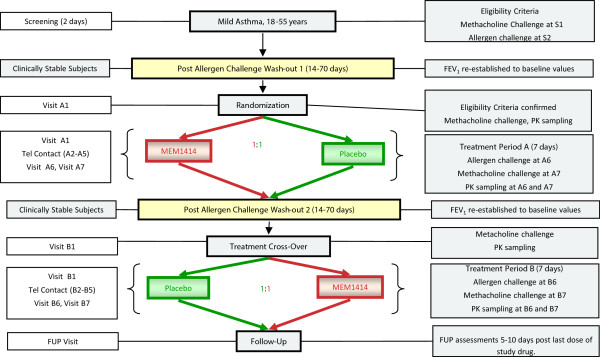
**Flow chart showing study design.** FeNO denotes exhaled nitric oxide. Allergen = inhaled allergen challenge. Methacholine = inhaled methacholine challenge.

This was a two centre double-blind, randomised, placebo controlled, cross-over study. Eligible subjects were randomised to receive MEM 1414 600 mg orally once daily (6x100 mg tablets) for 7 days (Figure 1). The washout period was 2–10 weeks between treatment periods. Dosing was performed under supervision at the sites on day 1, 6 and 7. On days 2–6 subjects were instructed to take the study medication at the same time of day, and were required to complete a diary card to document the time that medication was taken. Heart rate, blood pressure, ECGs, FEV_1_ and fractional exhaled nitric oxide (FeNO) were measured pre-dose and at 1 hour post-dose on days 1, 6 and 7. On day 6, an inhaled allergen challenge was subsequently performed after the 1 hour post-dose FEV_1_ and FeNO measurements. Methacholine challenge was then performed at 24 hours post allergen challenge. Adverse events and beta agonist use were monitored throughout the study with the aid of diary cards. A 6 day treatment period was considered sufficient to allow MEM 1414 to achieve steady state plasma concentrations. A minimum washout period of 2 weeks was specified after the first treatment period to allow allergic inflammation induced by allergen challenge to return to normal in these subjects. It also allowed adequate time to ensure that all active drug had been eliminated from the body and its effects had vanished before the following treatment period. For subject eligibility, FEV_1_ taken on day 1 of the first treatment period had to be within 15% of mean screen FEV_1_ (baseline). The day 1 FEV_1_ of treatment period 2 must also be within 15% of the FEV_1_ achieved on day 1 treatment period 1, if it was outside 15%, the subject was allowed to repeat the second treatment period day 1 visit within 10 weeks or was considered a withdrawal.

### Dose selection

Pre-clinical toxicology studies in rat and dog determined the NOAEL was 250 and 300 mg/kg/day respectively for both species. These oral doses produced plasma levels of 73 μg · hr/mL in rat and 116 μg · hr/mL in dogs. These studies were used to underwrite the selection of dose for clinical studies. Calculations using allometric scaling models suggested that the equivalent dose in humans would be 600 mg MEM 1414 with projected AUC 95 μg · hr/mL after 1 week dosing (data on file, Memory Pharmaceutical Corp.). In humans MEM 1414 has been studied in 5 clinical trials, in single ascending dose studies ranging from 15 mg to 1000 mg and in a 14 day multiple ascending dose trial ranging from 50 mg to 400 mg. Plasma concentration of MEM 1414 increased in a dose proportional manner. Adverse event data suggest MEM 1414 was well tolerated and no emesis reported.

### Allergen and methacholine challenges

Bronchial challenges were performed as we have previously described [17, 18] using a Mefar Dosimeter (Mefar,Bologna, Italy). Subjects were assessed for sensitivity to house dust mite, cat, grass pollen, and positive and negative controls by skin prick tests (Soluprick SQ, Alk Abelló (Reading, UK). The allergen for inhalation was selected according to the largest skin test wheal (positive >3 mm) and clinical history. Fresh solutions of allergen were made up in 0.9% saline in doubling concentrations from 250 SQ-U/ml to 32 000 SQ-U/ml. At screening, incremental doses of allergen were inhaled [17] until an early asthmatic response (EAR) was observed, defined as a fall in FEV_1_ of ≥20% from the post saline value, on at least one occasion, between 5 and 30 minutes after the final concentration of allergen. The late asthmatic response (LAR) was defined as a fall in FEV_1_ of ≥15% from the post saline value, on at least three occasions, two of which must be consecutive, between 4 and 10 hours after the final concentration of allergen. During the treatment periods, the total dose of allergen required to cause an EAR and LAR was administered as a single bolus dose. Subjects were administered doubling concentrations of methacholine from 0.03125 to 32 mg/ml until a ≥20% fall in FEV_1_ was achieved or the highest concentration of methacholine was administered. The provocative concentration required to reduce the FEV_1_ by 20% of the post-saline baseline value (PC20) was derived by linear interpolation between the lowest concentration that caused a >20% fall and the preceding concentration, as described [17].

### FeNO

Standardised FeNO measurements were performed using the NIOX® (Aerocrine, Solna, Sweden) with 3 valid tests being done to ensure reproducibility.

### Ex-vivo LPS/A23187 challenge

For each subject, a 5-point (including a vehicle control) MEM 1414 dose–response curve was constructed during the baseline assessment. This allowed a calculation of the percentage of inhibition of cytokine production for each specific subject during the treatment phase. Whole blood was diluted with supplemented RPMI and incubated with 4 concentrations of MEM 1414 and a vehicle control for 30 minutes at 37°C/5% CO_2_. Cells were then stimulated with increasing concentrations of either LPS (for subsequent TNFα, IL-6 measurement) or calcium ionophore A23187 (LTB_4_). Blood was processed as follows: blood was collected at each treatment period at the following times: post-dose/pre-allergen challenge and 3 and 8 hours post allergen challenge on day 6 and at 24 hours post-dose/post-challenge on day 7 for the *ex-vivo* measurement of the cytokines TNFα, IL-6 and LTB_4_. Whole blood was drawn into lithium-heparinized tubes, then transferred to plastic tubes containing heparin (50 U/ml) and kept at room temperature for a period no greater than 2 hours. Whole blood (200 μl) was pipetted into 96 well plates with 10 μl of either LPS (0.001 – 10 μM) or A23187 (0.1 – 20 μM) which were placed into a 37°C incubator in an atmosphere of 5% CO_2_ in air for 18 hours. Plates were then centrifuged (250x g for 10 minutes) and aliquots of plasma frozen (20°C) for assessment of cytokine responses. TNFα and IL-6 were measured by Luminex xMAP platform and commercially available assay kits from R & D systems (Abingdon, UK). LTB_4_ was analysed using stand-alone ELISA kit supplied by Assay Designs Inc (Exeter, UK).

### Pharmacokinetic sampling and bioanalytical method

On day 1 blood samples were collected pre-dose, and 1, 2 hours post-dose, and on day 6 pre-dose, and 0.5, 1, 1.5, 2, 4, 8, 12 hours post-dose, and on day 7 pre-dose. Plasma concentrations were analysed by liquid chromatography-tandem mass spectrometry.

### Statistics

Minimum LAR was derived as the minimum FEV_1_ value over 4–10 hours post-allergen challenge. Minimum EAR was derived as the minimum FEV_1_ over 0–2 hours post-allergen challenge. Weighted mean LAR and EAR endpoints were derived by calculating the AUC over the relevant time interval using the linear trapezoidal rule and dividing by the time interval. Twelve evaluable subjects were considered to sufficient to detect a difference of 50% in the LAR (area under the curve from 3–10 hours, AUC 3–10 h) between MEM 1414 and placebo with 80% power, assuming a within subject standard deviation of 50% and a 2-sided alpha significance level of 0.05 in a cross-over design. In order to achieve this, approximately 16 subjects were to be randomized to achieve a minimum of 12 evaluable subjects. The primary efficacy parameter is the LAR as defined by the area under the curve from 3–10 hours (AUC 3–10 h). This was calculated from the change in the observed FEV_1_ (L) compared to baseline (highest of pre-allergen challenge values) using the trapezoidal method. The EAR as defined by AUC 0–3 h was analysed in the same way as the LAR. Comparison between treatments for FEV_1_ AUC 3–10 h were carried out using an analysis of variance for a cross-over design (ANOVA) with centre, subject (sequence and subject within sequence), period and treatment as factors of the model. Statistical analysis was performed on the log 2-transformed values of the provocative concentration of methacholine required to produce a 20% reduction in FEV_1_ (PC20) to compare MEM 1414 with placebo. A mixed effects model was fitted with the factors treatment and period treated as fixed effects and subject as a random effect.

FeNO change from baseline ratio at all time points were analysed following a loge-transformation to compare MEM 1414 with placebo. A mixed effects model was fitted with the fixed effects period, treatment group, subject-level loge-transformed baseline, period-level loge-transformed baseline, planned relative time, treatment group by planned relative time interaction term and period-level loge-transformed baseline by planned relative time interaction term and the random effect subject.

*Ex-vivo* LPS challenge baseline assessments were compared by ANOVA followed by Dunnetts multiple comparison test. The concentration response data from treatment periods were plotted for each subject at each time point and the effective concentration to give the 95% maximal response (EC_max_) was derived from a sigmoidal regression was fitted to this data using the variable-slope four-parameter logistic dose–response model for each of the cytokines. MEM 1414 and placebo were compared by ANOVA on the EC_max_ change from pre-challenge.

Pharmacokinetic parameters were estimated using non-compartmental methods. Samples obtained from placebo subjects were all below limits of quantification.

## Results

Forty-seven subjects were screened and 16 suitable subjects identified who were randomized and completed the study; with 8 at each site. The mean age was 33 ± 7 years with a male:female ratio of 6:10. Mean BMI was 25 ± 4. Mean highest FEV_1_ and predicted were 3.2 ± 0.57 L and 94.89 ± 12.46% respectively. Mean FeNO was 61.82 ± 44.62.

### Allergen challenge

Each subject received the same allergen throughout the study, selected based on history and skin prick reaction to house dust mite (n = 10), grass pollen (n = 4) and cat (n = 2).

**Figure 2:**
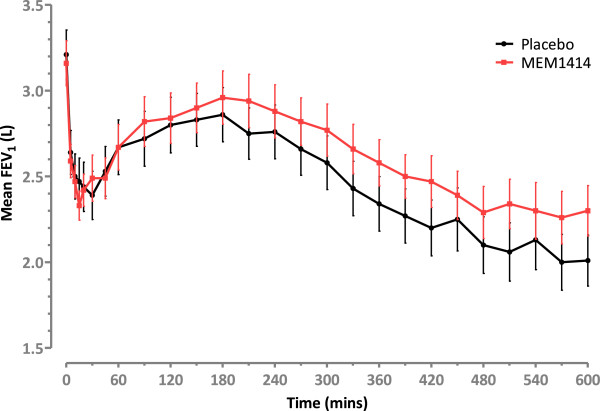
**Mean FEV**_**1**_ **over time.** Early and late asthmatic response to inhaled allergen challenge after 6 days treatment with either MEM 1414 or placebo. Means and 95% confidence intervals, change in FEV_1_ compared to post saline value shown.

**Figure 3:**
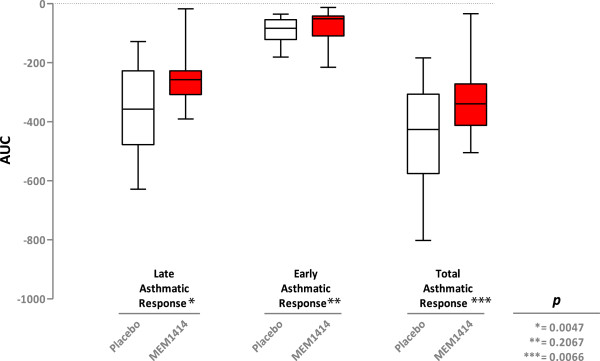
**Primary and secondary efficacy parameters.** Box whisker plot showing median, upper and lower quartiles. Whiskers represent the maximum and minimum values.

There was no significant period or sequence effect. Treatment with MEM 1414 abrogated the late phase response with a mean difference in FEV_1_ AUC (3-10 h) of 104 ml; this was a 25% reduction compared to placebo (p < 0.005), with no effect on the early response (Figures 2, and 3). The total asthmatic response as defined by FEV_1_ AUC 0-10 h also showed a significant difference between treatments (p = 0.0066).

### Methacholine PC_20_

The analysis used only the 11 subjects with complete data and the effect of treatment did not reach statistical significance. There was no significant period or sequence effect. The fold difference between the change in MEM 1414 and placebo from day 7 to day 1 was 1.34 mg/mL ±1.52 (geometric mean ± S.D., p = 0.38).

### Exhaled nitric oxide

**Figure 4:**
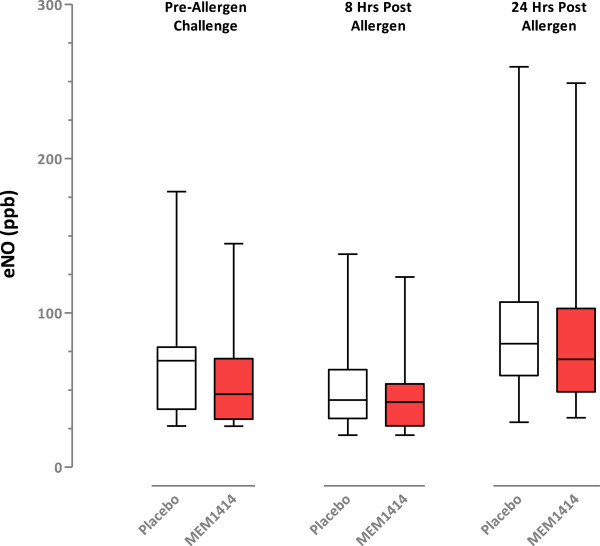
**Exhaled nitric oxide.** Box whisker plot showing median, upper and lower quartiles. Whiskers represent the maximum and minimum values.

The change from day 1 to day 6 post-dose/pre-challenge, and on day 6 at 8 hours post-challenge and day 7 at 24 hours post-challenge are shown in Figure 4. There were no differences between MEM 1414 and placebo. There were no significant period or sequence effects at any timepoints.

### Ex-vivo LPS/A23187 challenge

**Figure 5:**
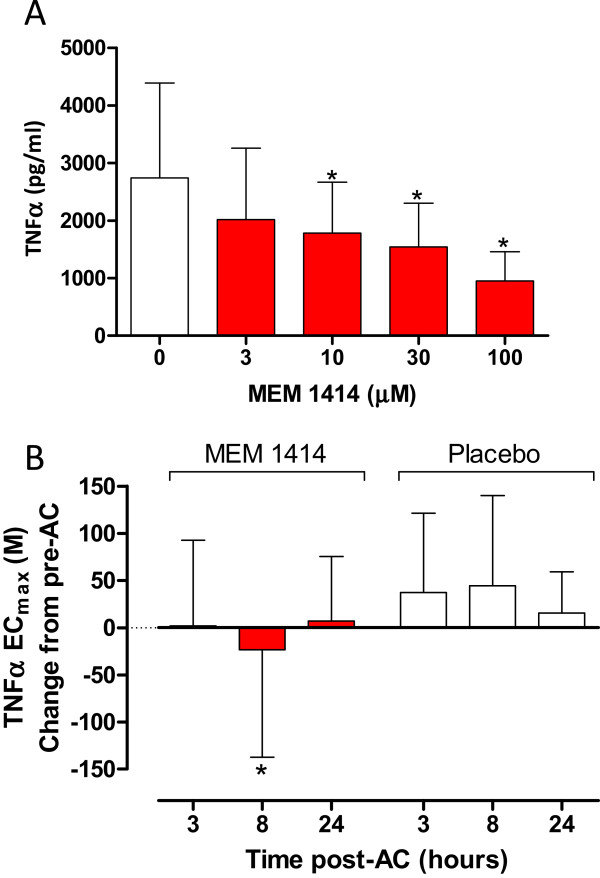
**TNFα release in stimulated whole blood.** **(A)** MEM 1414 concentration response curve baseline assessment for TNFα release in whole blood. Blood was stimulated with 1 μM LPS. *p < 0.05 ANOVA. **(B)** TNFα release in whole blood during treatment periods. Data shows change in EC_max_ TNFα release from pre allergen challenge compared to 3, 8 and 24 hours post allergen challenge. *p = 0.038 ANOVA. Data are mean ± S.D. AC: allergen challenge.

The baseline TNFα release from subjects is shown in Figure 5A. TNFα release was stimulated by the addition of 1 μM LPS. Data from IL-6 and LTB_4_ are not shown. A summary of the changes from pre- to post-allergen challenge are shown in Figure 5B for TNFα post dosing. At 8 hours post-allergen challenge there was a significant difference between MEM 1414 and placebo for the change in TNFα response from baseline (p < 0.038). There were no significant treatment effects seen in IL-6 (data not shown). In the LTB_4_ data at 24 hours post allergen challenge there was a trend to a difference between MEM 1414 and placebo change from baseline, however this was not significant (p = 0.0808;data not shown).

### Pharmacokinetics (PK)

Plasma concentrations of MEM 1414 were available in a total of 16 subjects. PK analysis of data following dose administration on day 6 is shown in Table 1. Plasma concentrations of MEM 1414 on day 1 and day 6 showed a T_max_ on day 6 at 1.9 h ±/-1.2 (mean ± S.D) and a C_max_ of 17000 ng/ml ±8500 with no evidence of accumulation using the 2 hour time point to compare the ratios at 1 and 2 hours on days 1 and 6 (ratio 1.0, at 1 h and 1.1, 2).

**Table 1:** **Summary statistics of PK analysis (ITT population)**

N = 16	C _max_ (ng/mL)	T _max_ (h)	AUCt (ng.h/mL)	AUC0-24 (ng.h/mL)	AUC (ng.h/mL)	λz (1/h)	t _1/2_ (h)
Mean	17000	1.9	87600	87600	89000	0.1313	6.8
SD	8500	1.2	47300	47300	33800	0.0995	2.5
Min	5530	0.9	26000	26000	41300	0.0740	1.8
Median	17600	1.7	84700	84700	85700	0.0881	7.9
Max	34300	4.6	201000	201000	151000	0.3849	9.4
GM	14800	1.6	75800	75800	83500	0.1120	6.2
CV%	50.0	63.2	54.0	54.0	38.0	75.8	36.8

### Adverse events (AEs)

Overall 14 subjects (87.5%) reported at least one AE in either the MEM 1414 or placebo treatment periods. More subjects reported AEs during treatment with MEM 1414 compared to during treatment with placebo (81.3% versus 56.3%), primarily due to a higher incidence of nausea, headache and diarrhoea during MEM 1414 treatment (Table 2). No subjects had an AE considered severe, no subjects were withdrawn due to an AE and no severe AEs were reported. The most common system organ classes with subjects reporting AEs were gastrointestinal disorders (10 subjects, 62.5%) and nervous system disorders (9 subjects, 56.3%). The majority of the AEs were mild (8 subjects [50.0%] reporting mild events only and 5 subjects [31.3%] reporting moderate events in the MEM 1414 treatment period and 5 subjects [31.3%] reporting mild events only and 4 subjects [25.0%] reporting moderate events in the placebo treatment period).

**Table 2:** **Summary of AEs reported in more than one subject (safety population)**

		MEM 1414	Placebo
		N = 16 (%)	N = 16 (%)
Subjects who reported any AEs		13 (81.3)	9 (56.3)
	Nausea	7 (43.8)	2 (12.5)
	Headache	6 (37.5)	2 (12.5)
	Diarrhoea	4 (25.0)	0
	Vomiting	3 (18.8)	0
	Dizziness	3 (18.8)	1 (6.3)
	Chest discomfort	2 (12.5)	2 (12.5)
	Dysgeusia	1 (6.3)	2 (12.5)

Gastrointestinal AEs were reported in a higher number of subjects following MEM 1414 treatment compared to placebo, with most of these events being considered drug-related by the investigators. There were no changes in any mean laboratory values over time.

## Discussion

This was a proof-of-concept study to determine the effect of MEM 1414 on allergen induced responses in mild asthmatics. There was a significant attenuation of the LAR in subjects treated with MEM 1414 which met the primary end-point of the study and this attenuation of the LAR is comparable in magnitude of effect to other oral and inhaled anti-inflammatory agents [9, 10, 17, 18]. For example oral roflumilast (IC_50_ 0.5 nM average for PDE4-A, -B and -D) and reduction in LAR AUC of 43% [10, 16] and inhaled inhibitors GSK256066 IC_50_ 0.0032 nM and a reduction in LAR of 34% [18, 19] and CHF6001 IC_50_ 0.026 nM with corresponding fall in LAR of 30% [20].

The allergen-induced response is a robust model that is commonly used to assess the therapeutic potential of novel treatments for asthma [9, 10, 17, 18, 21–26]. All of the currently available and effective treatments for asthma modify the LAR following allergen-induced responses and there are no false positives [27]. These medications include short-acting and long-acting inhaled β_2_-agonists, inhaled corticosteroids, cromones, methylxanthines, leukotriene inhibitors, and anti-IgE monoclonal antibody. Comparing the results of different allergen challenge studies requires caution, as methodological details such as the period of measurement of the late response can vary between studies (we measured up to 10 hours while some studies only measure up to 7 hours), and individual patient characteristics may differ. The magnitude of attenuation of the LAR is not directly comparable to the previous study involving the orally administered PDE4 inhibitor roflumilast, which inhibited the maximal fall in the EAR and LAR by 14-28% and 16-43% respectively [10, 28]. The level of inhibition of the LAR AUC of 25% seen in our study is similar to the results with an inhaled selective PDE4 with identical methodology [18] and greater than seen with IL-4 mutein pitrakinra (6% difference from placebo) by blocking the common receptor to IL-4 and IL-13 [22]. The lack of effect on the EAR may indicate that MEM 1414 has less effect on mast cell degranulation as seen with inhaled corticosteroids [21, 23, 24]. Previous studies with inhaled PDE4 inhibitors have shown mixed results on EAR, certain studies show an inhibition of EAR [10, 18, 28], while other studies show no effect on EAR [9, 19].

Following allergen challenge, mast cell activation is prolonged and leads to a more complex immune response – the LAR in certain selected asthma subjects [29]. The LAR involves T cell activation, influx of eosinophils and other inflammatory cells, with the release of cytokines and other inflammatory markers. Group 2 innate lymphoid cells (ILC2) are also important in the innate immune response by releasing large amounts of IL-13 and IL-5 in response to IL-33 from endothelial cells, and ILC2 cells play a role in the type 2 polarisation of T cells [30, 31]. PDE4 is expressed on cells involved in both innate immune response (mast cells) and the Th2 type inflammatory responses (eosinophils, macrophages and lymphocytes) [5, 26, 32]. PDE4 is also expressed on smooth muscle cells and has been shown to be involved in NANC neuronal inflammation in both guinea pig and human bronchus [33–35]. PDE4 inhibits both the inflammatory mechanisms and inhibits the regulatory networks that inhibit allergen responses. The LAR is therefore a well validated model to study inhibition of allergic inflammation, which supports the prediction of clinical efficacy.

The secondary endpoint measurements of methacholine challenge post allergen, or exhaled NO were unchanged by treatment. The study was not powered to show a difference in the secondary parameters and only 11 sets of paired data were obtained for methacholine challenge. This observation is often made in such studies as subjects lung function remain hyper-responsive post-allergen challenge and further procedures performed after this procedure such as methacholine challenge often cannot be performed for safety reasons.

Studies using inhaled corticosteroids have shown both attenuation [21, 25, 26] and no attenuation [36] of methacholine reactivity post allergen challenge. In line with these variable results, montelukast has also been shown to have no effect on methacholine reactivity post allergen challenge in one study [21] but an inhibitory effect in another [17]. These variable results suggest that methacholine reactivity post-allergen challenge is not a robust primary endpoint to evaluate drug effects.

Reducing FeNO levels by specific iNOS inhibition does not inhibit the EAR or LAR, suggesting that nitric oxide is not mechanistically involved in the pathophysiology of allergen-induced asthma [17]. However, FeNO is a sensitive biomarker and practical surrogate marker to monitor inhaled corticosteroid therapy [37–39]. Raised levels of FeNO are associated with inflammation in asthma and are sensitive to suppression by steroids and also associated with levels of asthma severity [40, 41]. The effects of the leukotriene receptor antagonist montelukast are more variable, with no inhibition observed of nitric oxide observed in some studies [17, 42]. The usefulness of FeNO as a biomarker appears to vary with the class of drug, and our results and others [18] suggest that airway nitric oxide production is a PDE4 independent mechanism. Alternative explanations are that the current study was too short or underpowered to detect a reduction in exhaled nitric oxide. Inhibition of TNFα release by PDE4 inhibition has been shown *in vivo* from monocytes, tracheal smooth muscle and recruitment of eosinophils to the airways following antigen challenge in immunized guinea-pigs and rabbits [43]. Roflumilast displayed a modest inhibition of LPS-induced TNFα release in subjects after 4 weeks treatment. TNFα release was inhibited by 1.3 fold in monocytes *ex vivo* [11]. Murine studies have shown that PDE4B is the main isoform for mediating the release of TNFα in response to LPS as well as other Th2 cytokines and eosinophil recruitment [44–46]. Monocytes, neutrophils, eosinophils and T lymphocytes almost exclusively express PDE4 and not any other PDE isoform [47, 48]. The effects of PDE4 on cellular function usually involve isolation and purification of the cell under investigation [49] or are performed in whole blood [43]. Inhibition of TNFα release from whole blood is consistent with findings observed with isolated cells, and further supports the mechanism of action of an anti-inflammatory effect for this class of agents [43].

Animal studies have suggested the potential benefit in the use of non-selective PDE4 inhibitors for targeting inflammatory cells compared with selective inhibitors of PDE4 isoforms. Recruitment of eosinophils in airway inflammation is no different in mice deficient in PDE4D than in wild type control mice, however in the PDE4 knock out mice, methacholine induced airway obstruction was eliminated [50]. This indicates that other PDE4 subtypes are involved in the metabolism of cAMP. PDE4B knock out mice do not undergo typical allergic inflammation responses such as Th2 cytokine production, eosinophil recruitment and bronchial hyperresponsiveness [45]. These two studies, amongst others, suggest a complementary role of PD4 isoforms in allergic airway inflammation and the need to target several PDE4 isoforms, especially as the inflammatory response is inhibited by non-selective PDE4 inhibitors such as rolipram and roflumilast [51].

There were few adverse effects in this study, but the incidence of gastro-intestinal side effects is comparable to that associated with other oral PDE4 inhibitors [13–15]. The pharmacokinetic analysis performed showed a C_max_ concentration of MEM 1414 at 1–2 hours post-dose was 17,000 ng/ml (40 μM).These plasma concentrations are much greater than the mean C_max_ of roflumilast administered orally (2,000 pg/ml) with good tolerability (although lower than the active metabolite roflumilast N-xide) [52] yet the *in vitro* IC_50_ values were similarly potent, with Ki values on purified human recombinant PDE4 isozymes (Ki 22, 18 and 12nM for the PDE4A, PDE4B and PDE4D isozymes; data on file, Memory Pharmaceuticals Corp.). Similar studies with V11294, and apremilast also showed good tolerability but also had lower concentrations (2–4000 ng/ml) [43, 49] In our study plasma concentrations at C_max_ were approximately ten fold higher than in studies with other PDE4 inhibitors and an absence of GI side effects [43, 49]. The selection of the dose for this study was empirical based on the maximum tolerated dose from previous studies in human volunteers and selected to ensure adequate PDE4 inhibition. The maximum tolerated dose is often selected for allergen challenge studies to increase the likelihood of success. Dose response studies are not commonly performed using the preferred cross over design in this type of study. It is often the case that doses selected for the first proof of concept study are too high and not necessarily relevant for subsequent clinical studies. Altering the pharmacokinetic properties of the formulation of MEM 1414 to avoid such high C_max_ concentrations of MEM 1414 may be a promising way to improve the side effect profile.

Inhibition of *ex-vivo* TNFα release were seen at sub-maximal LPS concentrations <16 ng/ml with both MEM 1414 and V11294 [43]. However TNFα inhibition in the present study was delayed to the 8 hour time point and not seen at t_max_ with concentrations when peak MEM 1414 concentrations were well in excess of the EC_50_*ex-vivo* (40 μM; 3x greater than EC_50_). A delay in equilibration to an active compartment may explain this delay in *ex-vivo* pharmacodynamics effect (Figure 5).

Oral PDE4 inhibitors have also been reported to show clinical efficacy in COPD patients [13–15], but with a significant rate of side effects. The information on the incidence of GI side effects for PDE4 inhibitors in asthma is less known. Although the current study using MEM 1414 was focused on asthma, using this drug in COPD would also be of interest.

## Conclusions

Oral MEM 1414 is a novel PDE4 inhibitor with nanomolar potency and high selectivity over other PDE isoforms. Treatment with MEM 1414 significantly reduces the late response following inhaled allergen challenge. MEM 1414 also inhibited whole blood assays of cytokine production from inflammatory cells. MEM 1414 was associated with a typical adverse event profile of PDE4 inhibitors, namely nausea and vomiting although these were mild side effects. Further development of MEM 1414 in inflammatory lung disorders such as asthma or COPD would be warranted given that a successful allergen challenge study has a useful predictive value in subsequent studies of clinical efficacy.

## Abbreviations

ANOVA: Analysis of variance
b.i.d.: Twice a day (bis in die)
BMI: Body mass index
cAMP: Adenosine monophosphate
cGMP: Cyclic guanosine monophosphate
Cmax: Maximum concentration
COPD: Chronic obstructive pulmonary disease
EAR: Early asthmatic response
FeNO: Fractional exhaled nitric oxide
FEV_1_: Forced expiratory volume in 1 second
FVC: Forced vital capacity
GI: Gastro-intestinal
GM: Geometric mean
IAC: Inhaled allergen challenge
ILC2: Group 2 innate lymphoid cells
LAR: Late asthmatic response
LPS: Lipopolysaccharide
LTB4: Leukotriene B4
NOAEL: No observable adverse events level
PC20: Provocative concentration to reduce the FEV_1_ by 20%
PDE: Phosphodiesterase
PK: Pharmacokinetics
S.D.: Standard deviation.
